# Labour diagnostics using the intrauterine pressure through sound waves emitted by a smartphone

**DOI:** 10.1007/s11517-025-03305-1

**Published:** 2025-01-31

**Authors:** Benjamin Alderson, A. Osman, Mahmoud Ahmed El-Sayed, Khamis Essa

**Affiliations:** 1 School of Engineering, University of Birmingham, Edgbaston, B15 2TT UK; 2 Marine and Offshore Engineering Department, College of Engineering and Technology, Arab Academy for Science and Technology and Maritime Transport, Alexandria, 21599 Egypt; 3 Industrial and Management Engineering Department, College of Engineering and Technology, Arab Academy for Science and Technology and Maritime Transport, Alexandria, 21599 Egypt

**Keywords:** Acoustic microscopy, Pressure sensing, Additive manufacturing, Obstetric diagnosis, Frequency analysis

## Abstract

Labour commencement diagnosis is still challenging in obstetrics. The majority of scientific techniques that were used to determine labour are costly and require a professional healthcare personnel to be carried out. Hence, in this work, an experiment was conducted using a 3D-printed 50% scale model of the abdomen of an average 40-week pregnant woman. The aim was to test whether the internal pressure can be evaluated from the reflection of the sound waves emitted by a smartphone. Frequencies of 4 kHz and 20 kHz were triggered at multiple distances (0.17, 0.34, 0.51 m) after inflating the 3D-printed model with water. The reflection coefficients and internal pressure were determined to have a positive linear correlation, suggesting that the hypothesis is practical. However, as the distances decreased, the reflection coefficient plateaued, indicating that the material had attained its maximum reflection coefficient at that frequency. Due to its reduced error and non-audible properties as compared to 4Hz, 20 kHz was suggested to be an optimum frequency for measuring pressure, allowing it for pain-free application for an extended amount of time.

**Graphical Abstract:**

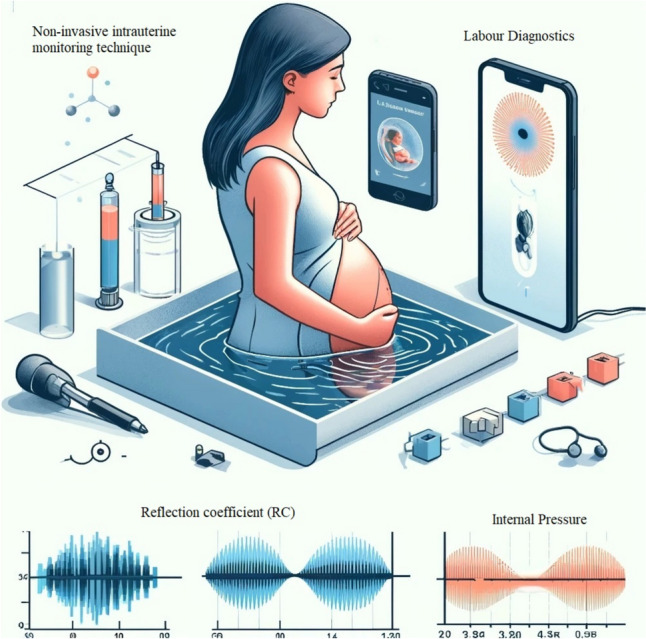

## Introduction

Labour diagnostics, particularly the accurate measurement of intrauterine pressure (IUP), is pivotal for safely delivering a new born. While there is no widely acknowledged conception of labour, three major contractions lasting 1 min within 10 min is a regularly used definition [1, 2]. A professional midwife or doctor commonly provides monitoring and diagnosis of the labour progress at a health centre; however, it could be conducted in-house if the woman is giving birth at home [3–5].

According to existing literature, many moms were reported to intensively rely on using mobile prenatal care apps as tools for health care management during pregnancy. However, such approach employed for assessing pregnancy may carry the risk of producing inaccurate and impractical evaluations [6, 7]. This obviously highlights the necessity for a method to evaluate the strength of a contraction without the usage of pricey healthcare technology or skilled medical professionals.

If intrauterine pressures can be measured at home by non-healthcare staff, using the widely accessible technology such as a cell phone, this would help assessing the labour and ultimately would boost the quality of care, time, and capacity management within the NHS [8–10]. It should be noted that the accurate measurement of intrauterine pressure (IUP) is essential for assessing labour progress, particularly in differentiating between strong and weak contractions. In clinical settings, a typical IUP during a contraction ranges from 30 mmHg (resting pressure) to 50–80 mmHg (during active labour), with strong contractions exceeding 50 mmHg [11–13]. Therefore, a pressure-measuring device with an accuracy of approximately ±10 mmHg would be sufficient to differentiate between weak and strong contractions, providing valuable insights into labour progression.

Sound wave propagation through biological tissues is influenced by the material properties of the tissue, specifically density and elasticity, which determine the acoustic impedance of each layer [14, 15]. The interaction of sound waves with tissue interfaces depends on the degree of impedance mismatch, which affects both reflection and transmission of the wave. The use of acoustic waves, particularly ultrasonic and sub-ultrasonic frequencies, has been extensively explored for various biomedical applications such as imaging, pressure sensing, and tissue characterization. These applications typically rely on the interaction of sound waves with the acoustic impedance mismatches at tissue boundaries. Earlier studies had employed invasive techniques to measure physiological pressures [16]. On the other hand, in a study by Vergilio et al. [17], high-frequency ultrasound (HFUS), which is a non-invasive imaging modality that utilizes sound waves, was applied to visualize skin structures. However, it required direct skin contact and the use of coupling gels to ensure effective transmission of sound waves. Other studies had applied non-invasive pressure sensing to investigate the propagation of low-frequency waves in closed systems, focusing on the correlation between wave reflection and internal pressure [18]. Nevertheless, most of these techniques either depend on invasive methods or require costly and bulky medical-grade equipment, which limits their use outside clinical settings.

Finite element method (FEM) is a powerful numerical tool for modelling the behaviour of sound waves as they interact with biological tissues, which have complex geometries and material properties [19]. FEM enables accurate simulation of how the propagated sound waves are absorbed, reflected, and transmitted through different layers of tissues with varying acoustic properties. This technique is particularly useful in medical acoustics where understanding of the interaction between sound waves and biological materials is critical for diagnostic applications [20–22].

Previous research has demonstrated that sound waves, particularly in the low kHz range, can be used to measure changes in internal pressure within a closed system. In addition, it was suggested that FEM could allow for a precise modelling of acoustic impedance mismatches between materials, which governs the reflection coefficient at the interface of different tissues such as skin, fat, and muscle. This is essential for analyzing how sound waves behave when they encounter these boundaries, particularly in complex biological structures. Earlier studies have successfully employed FEM to simulate sound wave propagation in diagnostic applications such as ultrasound imaging and acoustic pressure sensing [23]. By using FEM, it is expected to predict how sound waves could propagate through the abdominal wall and reflect back under varying internal pressures. The FEM model would account for the elasticity and density of biological tissues, enabling accurate predictions of how pressure variations affect sound wave reflection. This, in turn, would offer a novel non-invasive technique for monitoring intrauterine pressure.

In the current research, a novel acoustic pressure measurement approach using a smartphone was introduced, modelled, and experimentally validated. As a proxy for the uterus, a skin-like vessel was considered to study the effect of the internal pressure within such a container and the magnitude of sound reflected from the vessel outer wall surface. In this study, fused deposition modelling (FDM), a popular and relatively cheap additive manufacturing technique, was utilized [24, 25]. The process enables the fabrication of models with customizable mechanical properties [26, 27]. FDM was applied to manufacture the vessel mimicking the uterus, offering a reliable and accessible method for experimental replication. The FDM process allowed for a precise control of material porosity and stiffness, which are critical for simulating the acoustic impedance and reflection characteristics of biological tissues. This approach facilitated the reproducibility of experiments while maintaining cost efficiency.

In addition, due to its counterpart acoustical properties, polyurethane was selected to model the skin in this study. This might provide a proof-of-concept for the external measurement of intrauterine pressure. A model was created to demonstrate the change in internal pressure of a thin-walled vessel and its effect on the reflection coefficient of sound. Experiments were conducted via test rigs to validate the proposed principle. The interior pressure of a skin-like conduit that resembles the uterus was correlated with sound reflections. The results of such study would lead to the development of new approaches for health care management during pregnancy that are easier, cheaper, and more reliable for gravid women.

It should be emphasized that the method of using acoustic signals emitted by a smartphone was selected due to its cost-effectiveness, non-invasive nature, and wide availability. Traditional methods for measuring intrauterine pressure (IUP), such as intrauterine pressure catheters, are both invasive and expensive, limiting their use outside clinical settings. Moreover, these methods require professional healthcare personnel for operation, which can be inaccessible for home-based or resource-limited environments.

Regarding the use of sound waves in medical diagnostics, numerous studies have shown that sound waves, particularly ultrasonic and sub-ultrasonic frequencies, are effective for assessing tissue properties and internal pressures. Acoustic pressure sensing has been widely applied in medical diagnostics such as in assessing lung function, heart valves, and even for non-invasive fetal health monitoring [14, 28, 29]. The approach applied in this study uniquely integrates smartphone technology with sound waves to develop a cost-effective, non-invasive method for measuring intrauterine pressure (IUP). Unlike traditional ultrasound methods, this approach eliminates the need for direct skin contact, coupling gels, or specialized transducers.

Finally, additive manufacturing was chosen to create a realistic model of the pregnant abdomen for testing, as it allows for a high degree of customization and flexibility in material choice. By using fused deposition modelling (FDM) and a material like thermoplastic polyurethane (TPU), it was possible to mimic the skin’s mechanical properties, specifically its porosity and elasticity, both of which are important for accurately simulating the reflection of sound waves. The use of additive manufacturing to create a customizable model that emulates the characteristics of human skin further differentiates this work from prior studies, offering a replicable and scalable experimental setup.

## Methods

### Design and modelling

When the disparity in impedance between two materials is pronounced, sound is reflected upon striking the surface of the material with the higher impedance [21]. Hard surfaces are known to have significantly higher impedances compared to softer materials. The impedance contrast between air and skin is substantial, resulting in the majority of sound being reflected upon contact with the skin. To precisely replicate the acoustic properties of the skin, the material chosen for constructing the skin model in the simulation was a thermoplastic polyurethane (TPU) known as Flexfill 98a, supplied by Prusament [30]. This material was selected due to the possibility of obtaining a close acoustic similarity to the skin, as discussed later in this section.

The capability of a material to dissipate the energy contained in sound waves is influenced by the degree of porosity it possesses [31, 32]. This attribute is characterized by the fraction of void spaces within the material, with more solid objects having lower energy absorption capabilities and more porous materials having greater absorption properties [33]. Additionally, the elastic modulus and impedance of the material affect its ability to absorb sound, where materials that are pliant and flexible tend to have a greater capacity for sound absorption compared to rigid and stiff materials [34]. Consequently, in order to maintain close proximity to the properties of human skin in the model, maintaining a high degree of similarity in both the elastic modulus and impedance characteristics is essential, while also paying close attention to the level of porosity which is also a critical aspect that must be taken into consideration.

The porosity of human skin has been found to be approximately 15% with fluctuations observed depending on the specific bodily location [35]. Despite procuring information that was relevant to the replication of the abdomen, the data collected was derived from non-gestating females, thereby posing potential disparities between the actual porosity of a pregnant woman’s skin and the obtained measurements [36]. Further investigation would be required to ascertain whether these discrepancies exist.

To ensure that the experiment was both representative of the subject matter and cost-effective, the model skin was fabricated using an FDM 3D printing process with an 80% infill rate, with the aim of approximating the porosity characteristics of human skin. Despite the limitations in controlling and verifying the internal porosity of the thermoplastic polyurethane (TPU) material used, it was presumed that the resulting printed model exhibited a total porosity value of approximately 20%, which was deemed sufficient to simulate the properties of the skin.

The capacity of the skin to impede the transmission of sound waves is characterized by its impedance value, which is typically in the range of 1.7 × 10^6^ to 2.0 × 10^6^ *kg m*^−2^ *s* for healthy skin. This high level of impedance, which is several orders of magnitude greater than the impedance of air (4 × 10^2^ *kg m*^−2^ *s*), results in almost complete reflection of the incident sound waves (99.9%) with a negligible fraction absorbed upon striking the skin [20, 37, 38]. To ensure that the model skin effectively replicated the impedance properties of real skin, a thermoplastic polyurethane (TPU) material as its impedance value of 2.0 × 10^6^ *kg m*^−2^ *s* falls within the range exhibited by human skin [39, 40].

The skin’s elastic modulus exhibits a variability that is contingent upon the anatomical location within the human body, with values ranging from a low of 0.03 MPa to a maximum of 22 MPa [41]. Despite extensive investigation, there remains a paucity of literature that reports or provides data on the elastic modulus of the skin of pregnant women. An approximate value of 0.6 MPa for the elastic modulus of the skin on the abdomen of pregnant women has been estimated based on non-pregnant abdominal skin [41].

The elastic modulus of thermoplastic polyurethane (TPU), as a function of the manufacturing methodology and infill amount, can vary between a minimum of 0.1 MPa to a maximum of 50 MPa [40]. The TPU utilized in the study had an elastic modulus of 0.8 MPa with 80% infill, which is proximal to the value of the abdominal skin, thus making it an appropriate material choice. The reflection coefficient (RC) was calculated through the division of the reflected volume (Ir) by the incident volume (Ii) [37], as shown below:

1 $$\text{RC}=\frac{{I}_{\text{i}}}{{I}_{\text{r}}}$$

It should be noted that the ‘volume’ in this context refers to the sound intensity levels, measured in terms of pressure amplitude, rather than physical volume. The incident volume (*I*_i_) represents the initial sound intensity emitted from the source, while the reflected volume (*I*_r_) is the intensity of the sound wave after it reflects off the abdominal model surface. The following steps were applied for the measurement and calibration of the sound intensity and the subsequent data treatment;

1. Sound pressure level (SPL) measurement: Sound pressure levels were recorded using a calibrated microphone connected to a sound analyzer. The microphone was positioned at specific distances from the skin model to capture both incident and reflected sound waves accurately. The sound analyzer was set to measure SPL in decibels (dB), and these values were then converted to their linear equivalents for further analysis.
2. Data calibration: The incident sound pressure (*I*_i_) was first measured by recording the SPL at the source without any reflective surface. This value was used as a baseline for calibration. For the reflected pressure (*I*_r_), the SPL was recorded with the sound wave interacting with the model. A series of measurements were taken to account for environmental factors and ensure accuracy. Each measurement was repeated three times, and the average value was used to calculate the reflection coefficient.
3. Calculation of reflection coefficient (RC): The reflection coefficient (RC) was calculated using the formula shown in Eq. (1). The values of *I*_r_ and *I*_i_ were derived by integrating the area under the SPL curve over a specific time window to ensure that transient effects did not skew the results. The time window was chosen based on the duration required for the reflected wave to reach the microphone.
4. Normalization and error correction: To ensure consistency, all measurements were normalized against the baseline incident pressure. Additionally, corrections for background noise and environmental reflections were applied using a reference measurement taken under identical conditions without the abdominal model. This step was crucial for minimizing systematic errors and ensuring that the RC values truly represented the interaction between the sound wave and the model.
5. Software analysis: The collected SPL data was processed using Audacity software for volume and spectral analysis. The software allowed precise identification and extraction of the relevant frequency components. The extracted data was then analyzed in MATLAB to compute the RC and assess the impact of varying internal pressures on sound reflection.

The evaluation of shape change, as a function of applied pressure, is of particular interest, as it provides insight into whether the changes are significant enough to impact the RC, or whether the balloon is resisting pressure and not in complete contact with the skin. Even though the estimated changes in the dimensions of the skin model were small, the potential impact of these changes on the reflection coefficient must still be considered [37]. It is essential to assess whether these dimensional changes could influence the acoustic properties of the skin, such as its impedance and reflection characteristics. To precisely determine the effect of the skin dimensions on its acoustic properties, further analysis should be conducted to quantify the relationship between skin deformation and RC variations under different pressure conditions.

The dimensional changes mentioned in the study primarily refer to variations in the thickness of the skin layer and the overall profile changes of the belly under different internal pressures. These changes could include localized variations in skin thickness, shifts in the curvature of the abdominal surface, and the distribution of thickness across the belly’s profile. Skin thickness refers to the vertical measurement from the outer surface of the skin to the inner boundary (epidermis to dermis). Any variation in this thickness can change how sound waves are reflected or absorbed at the boundary, changing the skin acoustic impedance and affecting the RC values. Profile changes of the belly pertain to alterations in the overall shape and contour of the abdomen. Changes in the curvature of the abdominal wall, such as flattening or bulging due to varying internal pressures, can influence the path and interaction of sound waves, potentially affecting the RC. Thickness distribution describes how the thickness of the skin and underlying tissues is distributed along different sections of the abdominal profile. An uneven distribution may lead to localized variations in impedance, impacting how sound waves are reflected at specific points.

In this study, COMSOL Multiphysics software (COMSOL 6.2) was used to create a model to theoretically verify the veracity of the concept. The model can show if alterations in the internal pressure of a thin-walled container would result in a differing reflection coefficient even if the shape remains constant, as shown in [42–44].

In this study, porosity was used as a parameter to simulate how internal pressure variations affect the mechanical properties and reflection characteristics of the abdominal model. In biological tissues or models like the one used in the current study, internal pressure can affect the material’s structure. When the pressure inside the vessel increases, the material may expand slightly, increasing porosity. This change in porosity affects the material’s mechanical properties, such as stiffness and density. A decrease in porosity usually leads to an increase in stiffness and density, while an increase in porosity may make the material more compliant and less dense.

Porosity is expected to influence the acoustic impedance (the product of density and sound speed in the material). A higher porosity generally reduces the density and the effective stiffness of the material, which in turn lowers the acoustic impedance. Conversely, a lower porosity (denser material) increases impedance. In addition, the reflection coefficient is determined by the mismatch in acoustic impedance between two media (in this case, the material and air). When internal pressure changes the porosity, it alters the material’s impedance. This impedance change affects how much of the sound wave is reflected at the boundary versus how much is transmitted or absorbed.

As internal pressure changes, the model’s porosity and, consequently, its acoustic impedance are modified. These changes in impedance directly affect the reflection coefficient of the sound waves. For example, if increased pressure increases porosity (making the material lighter and decreasing its impedance), the reflection coefficient will decrease because of a greater mismatch between the impedance of the material and the surrounding air.

For modelling purposes, the relationship between pressure and porosity was established to mimic these real-world changes in tissue characteristics. By varying porosity, the model simulates how increased pressure could cause microstructural changes in the tissue, thereby affecting its overall acoustic response [45]. The introduction of porosity to the model was not done to artificially tune the model but to ensure that the simulation accounted for the impact of internal pressure on material properties.

To summarize, the key correlation in this study is that the internal pressure affects porosity, which in turn modifies the acoustic impedance of the material. This impedance variation influences the RC of sound waves at the material boundary. Therefore, monitoring changes in RC can provide insights into the internal pressure of the vessel, as the RC is sensitive to the pressure-induced porosity and impedance changes.

The model in this study was established as a two-dimensional representation of an average 40-week pregnant abdomen, whose dimensions were drawn from prior literature and were quantified to be 310 mm in width and 170 mm in depth [46–48]. To take advantage of the symmetry inherent in the abdominal structure and to minimize computational requirements, a half-body approach was adopted for the model’s construction. The thickness of the skin was modelled to be 3 mm, with additional fat and muscle layers incorporated into the model and set at 10 mm and 30 mm, respectively, based on the average values obtained from a cross-section of expectant mothers [49–52].

The interior of the simulation was depicted as a pressurized water-filled vessel, to imitate the uterus in the model. The boundaries were established as artificial limitations, simulating an open room environment, with a centrally located point source positioned at a specified distance from the model. The concept of porosity, a dimensionless metric quantifying the volumetric proportion of voids within a material relative to its total mass volume, was integrated into the simulation with a range of values encompassing 0.1 to 0.6, adhering to the reasonable limitations of material porosity [53, 54].

Earlier studies had suggested that frequencies in the range from 2 to 20 kHz are appropriate for non-invasive pressure measurement because they balance wave penetration with reflection properties. Sound waves within this frequency range would also have the ability to interact with the skin and underlying soft tissues without requiring direct contact (e.g., via ultrasound gel) [20, 55]. Therefore, this range was considered suitable for modelling soft tissue behaviour, such as in pregnant women’s abdomens, without significant wave dissipation. In the current study to thoroughly investigate the occurrence of resonant phenomena over a broad frequency range, the simulation was executed over a frequency spectrum spanning from 2 to 20 kHz.

To evaluate the sensitivity of the simulation to variations in the skin’s acoustic impedance, a parameter sweep was conducted. Skin impedance is known to vary depending on factors such as porosity, hydration, and anatomical location. The typical impedance range for healthy skin is between 1.7 × 10⁶ and 2.0 × 10⁶ kg/m^2^s. This variation could be further exaggerated in pregnant women due to changes in skin elasticity and porosity during pregnancy [38]. Using FEM, the parameter sweep was conducted by incrementally adjusting the acoustic impedance within this range and measuring the resulting RC over a range of frequencies from 2 to 20 kHz sound waves. The internal pressure of the uterine model was varied during the simulations to simulate contractions, while the skin impedance was altered to observe its effect on the sound wave reflection.

Finally, in order to experimentally validate the simulation results, a skin-like container was considered, as an analogy for the uterus. Experiments were carried out to investigate the effect of the internal pressure within such a container and the magnitude of sound reflected from its outer wall surface, as will be detailed in the following sections.

### Fabrication

**Fig. 1:**
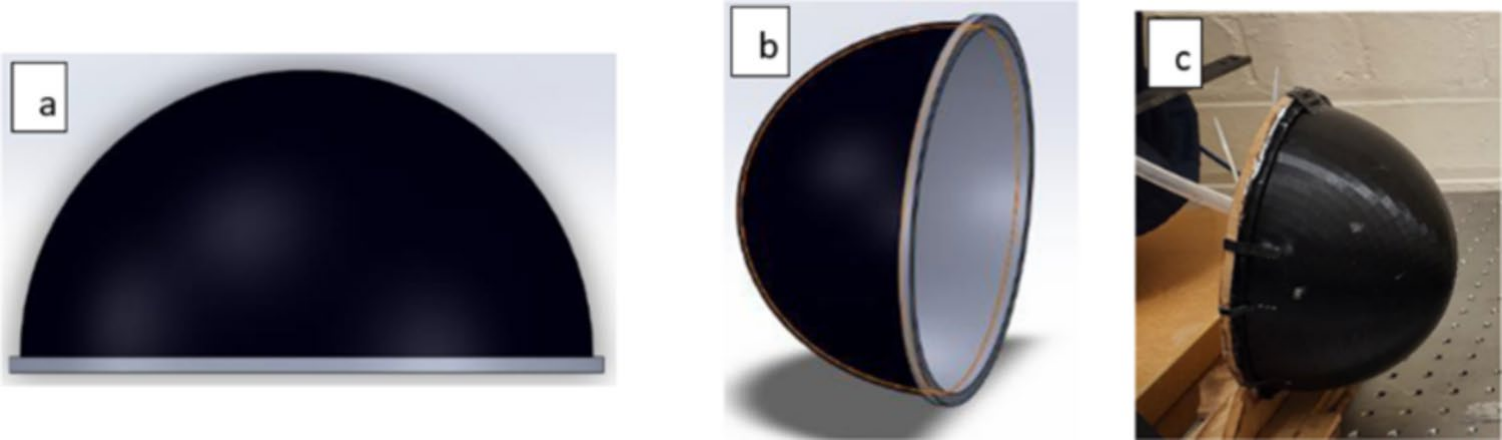
**a** Side view and **b** rear view of the CAD model of the skin. **c** 3D-printed skin. The width, depth, and thickness of the part are 310, 170, and 3 mm, respectively

The exoskeleton of the object under consideration was fabricated utilizing Flexfill 98a Prusament filament, a material widely used in FDM processes for several applications. The exterior casing of the skin model was designed with a thickness of 3 mm, which falls within the range of typical skin thickness found in the abdominal region in pregnant women, as described above. It was crafted to resemble human skin in terms of the material’s acoustic properties, thickness profile, and surface texture. This was essential for ensuring that the experimental setup accurately mimicked real-world conditions. This allowed the reflection coefficient measurements to be as realistic as possible, helping to validate the feasibility of using sound waves to measure intrauterine pressure. The CAD model is presented in Fig. 1a and b, while the fabricated part is shown in Fig. 1c.

The printing process was executed through the utilization of PrusaSlicer, a cutting-edge software that enabled the generation of a sliced model and accompanying G-code. The part was fabricated using a layer thickness of 0.2 mm and an infill density of 80. The model of the skin in the current study was designed with a dome-like shape, simulating the curvature of a pregnant abdomen. To enhance the stability of the object, the widest point of the skin was employed as the base, as depicted in Fig. 1a. The ‘widest point’ refers to the area of maximum curvature in the horizontal plane, which was used as the base for stability during testing. This point is where the skin model has its largest diameter, though the overall thickness of the skin remains constant at 3 mm. In addition, a central support structure was implemented to provide the necessary reinforcement for the overhanging dome of the skin. In this way, the model was expected to provide the necessary mechanical robustness and dimensional accuracy and accordingly could met the required aesthetic and functional properties for simulating human skin in the experimental setup.

The rear support panel was crafted from medium density fibreboard (MDF), while the inflatable component located within the integument was fabricated from latex. The size of the balloon was intentionally exaggerated in relation to the skin to prevent any potential influences on the generated pressure as a result of the elastic properties of the latex, serving merely as a barrier and container for the liquid.

The behaviour of a sound wave, whether it is reflected or penetrates, is dependent upon the difference in impedance between the mediums through which it is propagating. With an acoustic impedance approximately four orders of magnitude higher than air, the integument reflects over 99% of any incident wave [37]. Hence, ultrasound scans are performed with direct contact between the emitter and skin to facilitate penetration of the waves rather than reflection at the surface [20, 38].

Modelling the underlying adipose tissue and musculature was deemed unnecessary as they do not influence the reflected sound but rather maintain the structural integrity of the skin. Hence, it was deemed sufficient to imitate only the integumental layer for the purpose of accurate representation of the actual scenario. The Flexfill material, with a thickness of 3 mm, was found to possess the requisite durability to withstand the pressure and weight of the water when secured in place, thereby simplifying the assembly process and facilitating easier sealing of the skin. To accomplish this, a 3-mm hole was drilled into the wooden panel, and the balloon was inserted into the integumental cavity along with a piece of 3-mm-diameter plastic tubing, resulting in an effective seal via an interference fit and allowing pressurization of the skin through the filling of water through the plastic tubing.

### Experimental setup

An experimental investigation was conducted to examine the correlation between the simulated internal pressure within a mockup, designed to mimic the skin and intra-abdominal pressure conditions of a pregnant abdomen, and the RC of sound waves. The mockup was used to investigate how changes in internal pressure affect the reflection of sound waves, simulating the intrauterine pressure (IUP) during uterine contractions.

**Fig. 2:**
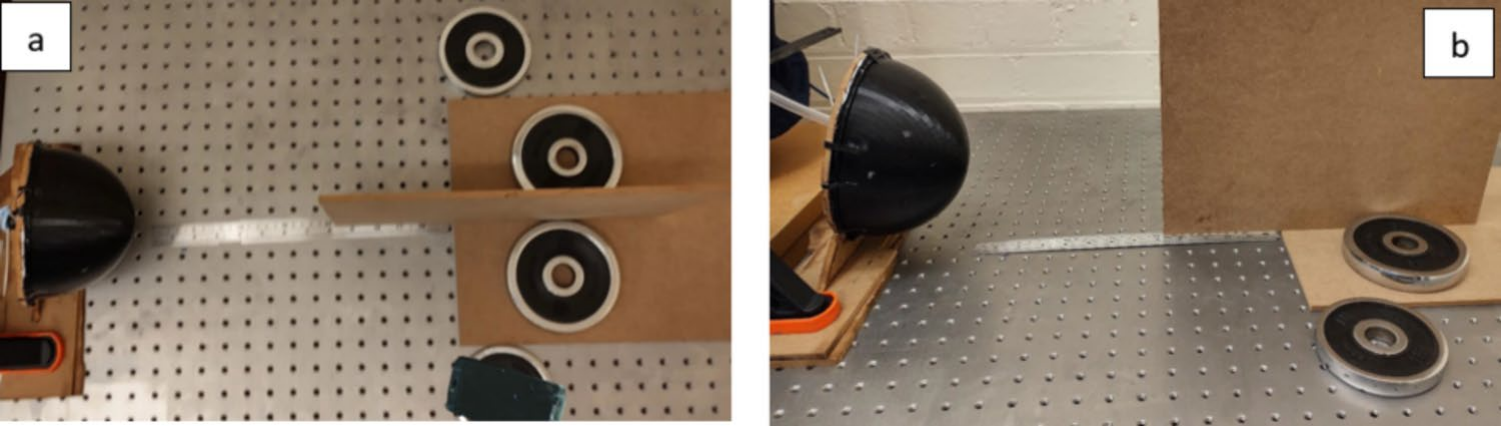
**a** Top-down view and **b** side view of the experimental setup showing the placement of the smartphone used to emit sound waves towards the abdominal model. The position of the second smartphone, used for receiving and recording reflected sound waves, is not depicted in this image but was positioned symmetrically on the opposite side of the model to ensure consistent data capture

In order to achieve adequate structural integrity and durability during the experiment, the cutaneous specimen was firmly secured to a rigid wooden platform to facilitate the necessary sealing and adhesion procedures. Furthermore, an inflatable balloon was inserted within the skin, and a perforation was meticulously created at the posterior aspect to permit the traversal of the balloon and the conduit, which were subsequently sealed to prevent any loss of fluid or experimental material. The experimental methodology utilized the principles of hydrostatic pressure to simulate the pressure associated with a uterine contraction. By modifying the height of water in predetermined, measured increments, different levels of pressure could be generated and evaluated. Detailed schematics of the experimental configuration can be found in Fig. 2a and b.

In the context of this study, two handheld electronic devices, specifically iPhone 12 (iOS 15.6) and Samsung Galaxy S21 (Android 12), were used in the experiment. The iPhone 12 features an integrated microphone with a frequency response range of 20 Hz to 20 kHz and a speaker capable of emitting sound at up to 24-bit/48 kHz audio quality. The Samsung Galaxy S21 is equipped with a microphone with a similar frequency response and a speaker that supports 24-bit/192 kHz audio playback. Both devices were configured to emit and receive sound waves, with recording settings adjusted to capture audio in lossless format to ensure high fidelity. These specifications ensure that the devices can accurately emit and detect the sound waves used in the study.

To ensure the accuracy and reproducibility of the frequencies emitted by the smartphones (iPhone 12 and Samsung Galaxy S21), the built-in speakers were benchmarked against a laboratory-grade signal analyzer (model: Agilent 35670A). The emitted frequencies were recorded using an external calibrated microphone connected to the signal analyzer. Any deviations in the emitted frequencies were quantified, with results showing a maximum variation of ±0.1 kHz at 20 kHz, which falls within an acceptable margin for this study.

The devices were strategically positioned at an equal distance relative to the central axis of the cutaneous surface, in such a manner that the incident angle with respect to the skin surface was fixed at 20°. Specifically, one of the devices served the purpose of a microphone, while the other device functioned as a speaker that was used to produce acoustic stimuli of interest at frequencies of 4 kHz and 20 kHz, as stipulated by the experimental design [56].

The sound waves used in the experiment were generated using a mobile application called “Frequency Sound Generator”. While this app allows for the generation of a range of frequencies, it is not laboratory-grade equipment and does not provide detailed technical specifications or calibration data. Therefore, the application’s emitted sound waves were compared to a reference-grade signal generator (model: Keysight 33220A) to ensure consistency. The output frequencies were recorded and analyzed, showing an error margin of ±0.05 kHz across the frequency range of interest (2 to 20 kHz). However, this might still represent a limitation that could affect the precision and reproducibility of the experiment. It is highly recommended, as a future research work, to employ calibrated laboratory equipment to generate and measure sound waves, ensuring greater accuracy and reproducibility of the results.

**Fig. 3:**
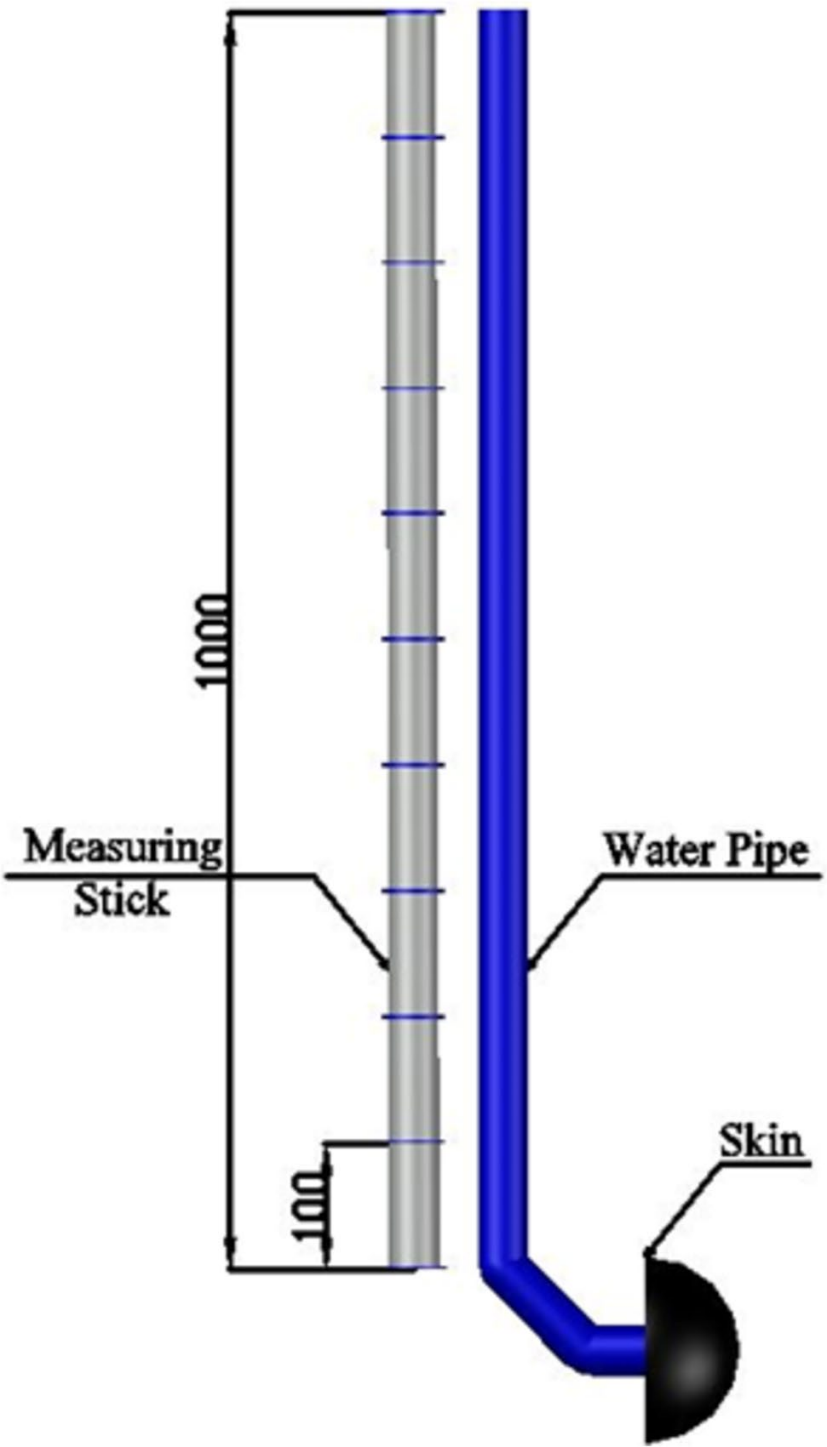
Water pressure system and measuring tool

The experimental setup involved the installation of a duct to the posterior aspect of the cutaneous specimen, which was then raised vertically to generate the desired pressure gradient. A rigid plastic component was utilized to fasten the duct, which was clearly demarcated with calibration marks at precisely measured intervals of 100 mm. To derive the pressure values, water was gradually introduced into the duct until it reached the designated mark, which was meticulously gauged using the markings on the plastic component, as illustrated in Fig. 3. Subsequently, this allowed for the calculation of pressure values using the fundamental principles of hydrostatic pressure, *P* = *ρgh*, where *ρ* is the density, *g* is the gravity acceleration, and *h* is the height. The height of water is varied allowing accurate pressure readings to be generated.

The pressure range selected for this study, up to 80 mmHg (10.67 kPa), is consistent with typical intrauterine pressure values observed during labour contractions. Normal uterine contractions in active labour generally range from 30 to 50 mmHg (4 to 6.67 kPa) but can reach up to 70 to 80 mmHg (9.33 to 10.67 kPa) during the peak of contractions [11–13]. This makes the chosen upper limit of 80 mmHg realistic and relevant for modelling the conditions experienced during labour.

The step increments of 100 mmH₂O (approximately 0.98 kPa) allow for a detailed exploration of the relationship between pressure changes and the reflection coefficient (RC) of sound waves. These increments are small enough to capture subtle variations in wave behaviour, yet large enough to distinguish meaningful differences in pressure reflection patterns.

The experimental setup entailed the formation of a triangular configuration with the skin serving as the apex, while the speaker and microphone were strategically positioned on opposing planes of the base. Both the speaker and the microphone were elevated to a height that corresponded to the central point of the skin, estimated to be 123 mm above the ground. To minimize potential confounding effects, a partition was erected between the source and receiver to isolate the test area from external acoustic interference. The partition was constructed from MDF board, with dimensions of 2.4 m/1.2 m × 22 mm. The material was chosen for its ability to attenuate sound in the 4–20 kHz range, as it has a high sound transmission class (STC) rating and is effective at reducing airborne noise. The partition was positioned perpendicular to the sound source at a distance of 1.2 from the setup, extending beyond the area of direct sound propagation paths to minimize diffraction effects. The material’s acoustic properties, particularly its density and thickness, were selected to reduce sound wave transmission and reflections that could interfere with the experiment. This is illustrated in Fig. 2.

The chosen material’s effectiveness at the tested frequencies (2–20 kHz) was evaluated by measuring background noise levels and reflected sound waves with and without the partition in place. Preliminary tests confirmed a significant reduction in background interference and unwanted reflections, particularly in the 10–20 kHz range, where the partition provided over 30 dB of attenuation. However, its performance was slightly less effective at lower frequencies (around 4 kHz), with an attenuation of approximately 15 dB. To validate the partition’s effectiveness, measurements of background noise and reflected signals were conducted both with and without the partition. The results showed a clear reduction in ambient noise and reflected sound interference when the partition was used, confirming its utility in isolating the test environment and ensuring the accuracy of the reflection coefficient measurements. However, it should be noted that while the partition significantly reduced interference, a complete isolation in this frequency range is challenging with simple panel materials. Therefore, results should be interpreted with an understanding of these limitations, and further refinements could include using more specialized acoustic barriers or anechoic chambers for future experiments.

The cutaneous specimen was thoroughly filled with water, and extra precaution was taken to ensure that all air was completely expelled to prevent any potential deviation in pressure readings. A baseline reading was established when the water level reached the top of the skin surface. Subsequently, a comprehensive frequency sweep was conducted with the cutaneous specimen entirely inundated with water. During the test, the pressure values were maintained at the reference baseline level, which corresponds to atmospheric pressure (approximately 101.3 kPa or 0-gauge pressure). This baseline was used as a control condition to ensure that any variations in the reflection coefficient were due solely to changes in internal pressure above this reference point.

A frequency sweep analysis was conducted over the range of 2 to 20 kHz. The background noise evaluation, assessment of the signal-to-noise ratio (SNR), and reflection coefficient (RC) measurements were taken at 1 kHz intervals. The results elucidated a stable SNR across all tested positions. In addition, they indicated that the RC values had peaked at 4 kHz, demonstrating the highest response of the specimen at this frequency. The data showed a clear trend with a marked increase in RC at 4 kHz compared to other frequencies. This observation suggests that 4 kHz is the most effective frequency for maximizing sound wave interaction with the cutaneous model in the context of this study. Hence, this specific frequency was considered the most appropriate option for subsequent experimental tests, primarily due to its unique characteristic of being easily discernible from other sources of background noise and its substantially enhanced peak amplitude, which ensured a more accurate and detectable outcome. To augment the study’s comprehensiveness and broaden its scope, additional experiments were conducted using a frequency of 20 kHz, with the intent of elucidating the potential effects of high-frequency soundwaves on the cutaneous tissue, while simultaneously providing comparable data to the 4 kHz experimental outcomes.

Subsequent to the selection of 4 kHz as the optimal frequency, a series of experiments was performed, whereby this frequency was played for a duration of 20 s, and the process was repeated five times. Subsequently, the distance between the speaker and microphone to the skin was systematically reduced, with the sequence of measurements being repeated three times at various distances, for each water height, with 0.1 m intervals ranging from 0.0 to 1.0 m. To ensure the consistency and accuracy of the measurements, the dimensions of the cutaneous specimen, specifically the skin depth and width, were recorded meticulously at every incremental height. This was to ensure that the internal pressure was being transmitted correctly to the skin model. The measurements were taken using digital callipers with a resolution of 0.01 mm, which provided precise and accurate readings of the specimen’s dimensions under varying pressure levels. This allowed monitoring any expansion or deformation of the model during the experiment.

**Fig. 4:**
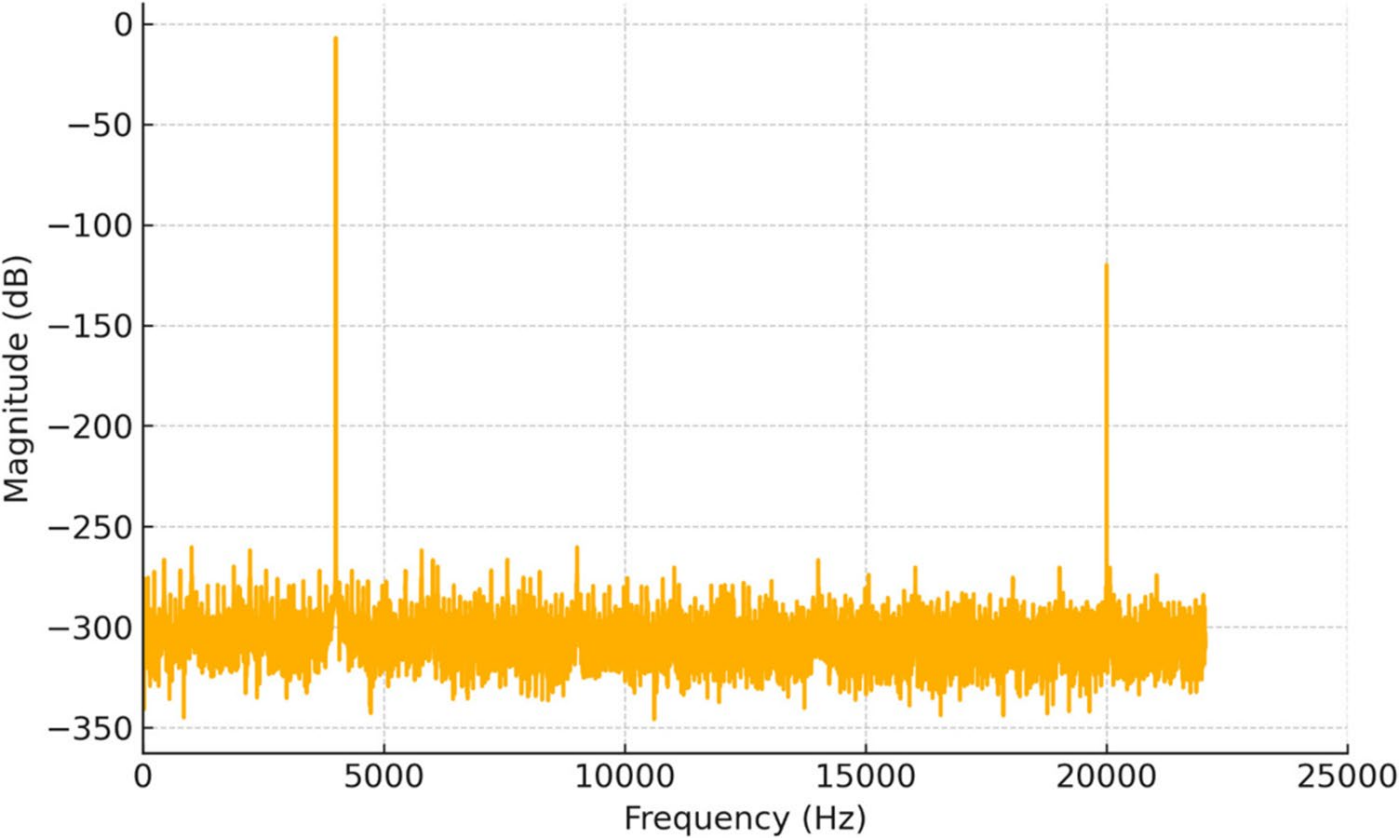
FFT spectrum for 4 kHz and 20 kHz frequencies

In order to extract the requisite data, a series of sequential steps were conducted, as depicted in Fig. 4. The software Audacity (version 3.0.2), developed by The Audacity Team and available under the GNU General Public License (GPL), was used for volumetric and spectral analysis of the recorded sound files. Audacity was chosen for its open-source nature, compatibility with various audio formats, and ability to provide accurate frequency analysis necessary for the experiment. The raw sound file was loaded into the software, for analysis. These analyses were meticulously conducted, and the resulting frequency spectrum was carefully examined to ensure accuracy. Upon completion of the analysis, the frequency spectrum was exported to a document, where the corresponding frequency values of interest were carefully identified and precisely highlighted, in accordance with the experimental design. The identified frequency values were then transferred to a spreadsheet application, where subsequent statistical analyses were conducted to further elucidate the data.

Based on the provided data for the two key frequencies (4 kHz and 20 kHz) used for experimental testing, the Fourier transform (FTT) for the loudness changes as a function of frequency was plotted. It was assumed that the waveform is a combination of sine waves at 4 kHz and 20 kHz, and a simulated plot was generated for these two frequencies. This is shown in Fig. 4. This plot represents the FFT spectrum of a waveform containing two key frequencies: 4 kHz and 20 kHz. The plot shows distinct peaks at both frequencies, with a much lower amplitude for the 20 kHz signal (as specified by the −120 dBFS loudness) compared to the stronger 4 kHz signal (−7 dBFS). This simulation aligns with the provided experimental conditions and highlights the difference between audible (4 kHz) and ultrasonic (20 kHz) frequencies.

The values and ranges of the experimental variables used during this study are given in Table 1, with the relative loudness of sound (measured in decibels) being a dependent variable. Other variables including the frequency, height of water, and distance from skin were considered independent variables. Water height was varied to simulate different levels of intrauterine pressure, with higher water heights corresponding to greater internal pressures. This approach allowed for a controlled investigation of the relationship between pressure and reflection coefficient (RC). The distances between the smartphone and the skin model were selected to replicate practical usage scenarios, ensuring the feasibility of non-invasive monitoring under realistic conditions. Frequencies of 4 kHz and 20 kHz were chosen to compare sound wave behaviour across both audible and ultrasonic ranges, highlighting the advantages of 20 kHz for precision monitoring and its minimal susceptibility to interference. The dependent variable was then systematically measured and recorded under different combinations of the independent variables. The selection of these variables was crucial in enabling a comprehensive and informative comparison of the pressure measurements under diverse experimental conditions.

**Table 1:** Summary of experimental variables and conditions, including frequency, water height, and distance from skin

Experimental conditions	Value
Frequency (kHz)	4, 20
Relative loudness of sound (dBFS)	−7.0 to −120.0
Height of water (m)	0.0 to 1.0
Distance from skin (m)	0.17, 0.34, 0.51
Skin width (m)	0.145 to 0.151
Skin depth (m)	0.105 to 0.109

The selection of 20 kHz and 4 kHz frequencies for experimental testing was based on a deliberate consideration of their specific attributes. Specifically, 20 kHz was chosen for its ability to facilitate “silent” measurements, given that it lies beyond the range of human hearing, and for the potential insights, it could offer on the performance of ultrasonic frequencies. However, the 4 kHz frequency was selected as the most promising option based on the outcomes of the frequency sweep. The exclusion of additional frequencies was mainly due to practical constraints, such as the time limitations inherent in conducting the experiment and the scope of the research, which was primarily centred on proving the conceptual feasibility rather than developing a fully functional prototype. The manipulation of the water height was designed to simulate a range of intrauterine pressures (IUP) that would vary from 20 to 80 mmHg, as described above.

In the current experiments, the height of the water was adjusted incrementally to create a corresponding pressure range that mimicked these physiological conditions. In this manner, it was possible to replicate these pressures to ensure that the model closely aligned with real-world uterine conditions during labour. By selecting these values, it was aimed to ensure that the experimental setup accurately represented the physiological range of IUP, allowing for a realistic simulation of resting and contracting uterine states.

Similarly, the variation of the distance from the skin served to explore the potential effects on the resolution and quality of the results. This enabled the investigation of whether distance had an impact on the rectified RC or the absolute volume recorded.

To ensure reproducibility, all experiments were repeated three times at each parameter setting, and the results were averaged to minimize variability. In addition, in order to validate the results, additional testing was conducted using a single device as both the speaker and microphone, with the device being placed directly opposite the centre of the skin. The other experimental parameters remained the same, with the divide being replaced by the phone. This setup would allow to verify the RC measuring results by eliminating any potential discrepancies introduced by using separate devices for emitting and recording sound.

## Results

**Fig. 5:**
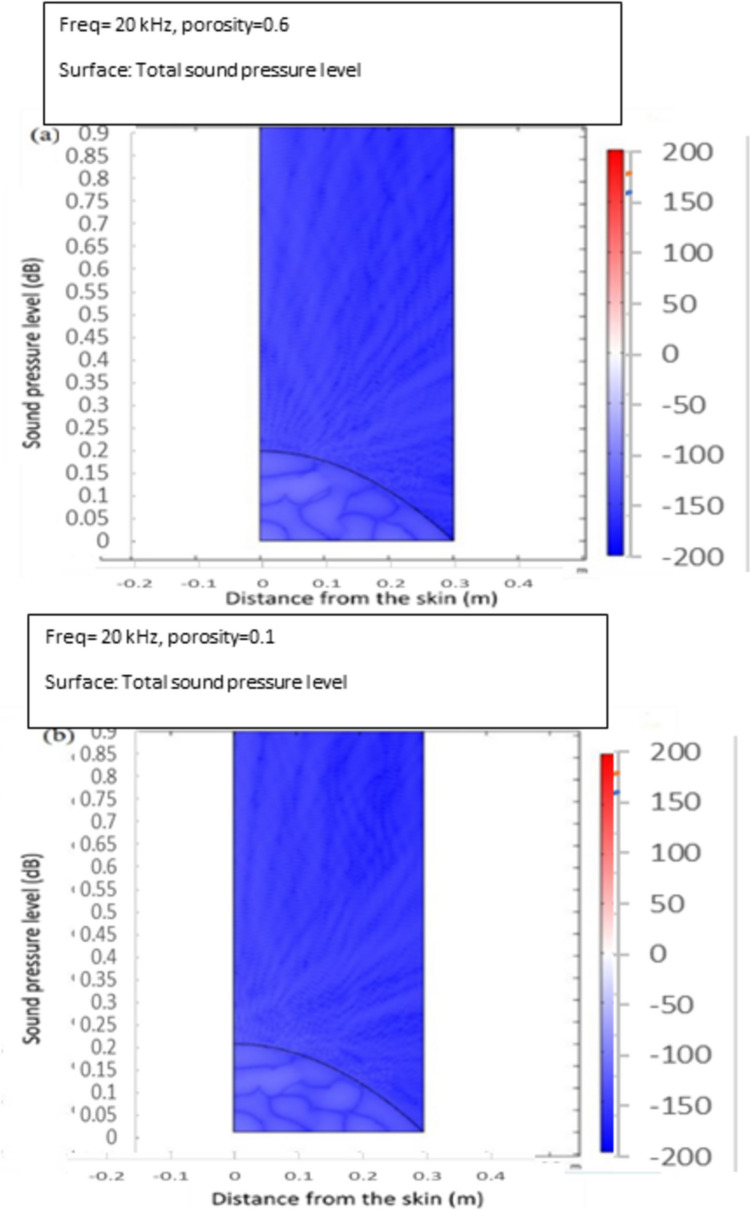
Impact of porosity levels on reflection coefficient (RC) at a constant frequency of 20 kHz under varying pressure conditions: **a** 0.6 and **b** 0.1 porosity levels

The modelling results representing the reflection coefficient variation with different sound pressure levels, distance from the skin, and porosity levels are presented in Fig. 5a and b. Simulation results demonstrated that varying porosities and the subsequent pressure changes, while maintaining constant geometry, had resulted in distinct reflection coefficients (RCs). The effect of porosity could be detected by comparing RC values at 0.6 and 0.1 porosity levels. At a higher porosity level (0.6), the material shows increased sound absorption, leading to lower RC values. Conversely, at a lower porosity level (0.1), the material exhibits higher stiffness and acoustic impedance, resulting in higher RC values. Regarding the pressure effect, although subtle in the figure, as internal pressure increases, the material stiffens due to the compression of voids, reducing sound absorption and increasing RC. This trend is evident for both porosity levels, with RC values converging slightly at higher pressures. This behaviour aligns with the expected response of biological tissues, where changes in porosity due to pressure significantly influence acoustic properties. These results could be considered a validation of the concept and therefore provided the basis for proceeding with a physical experiment.

The results of the parameter sweep showed a noticeable sensitivity of the RC to changes in skin impedance. As the impedance increased towards 2.0 × 10⁶ kg/m^2^s, the RC values increased, indicating stronger reflections. The simulation results confirmed a positive linear relationship between impedance and reflection coefficient, which suggests that variations in skin impedance significantly impact the accuracy of intrauterine pressure measurements via sound waves. For lower impedance values (closer to 1.7 × 10⁶ kg/m^2^s), there was greater sound wave absorption by the skin, which led to lower RC values.

**Fig. 6:**
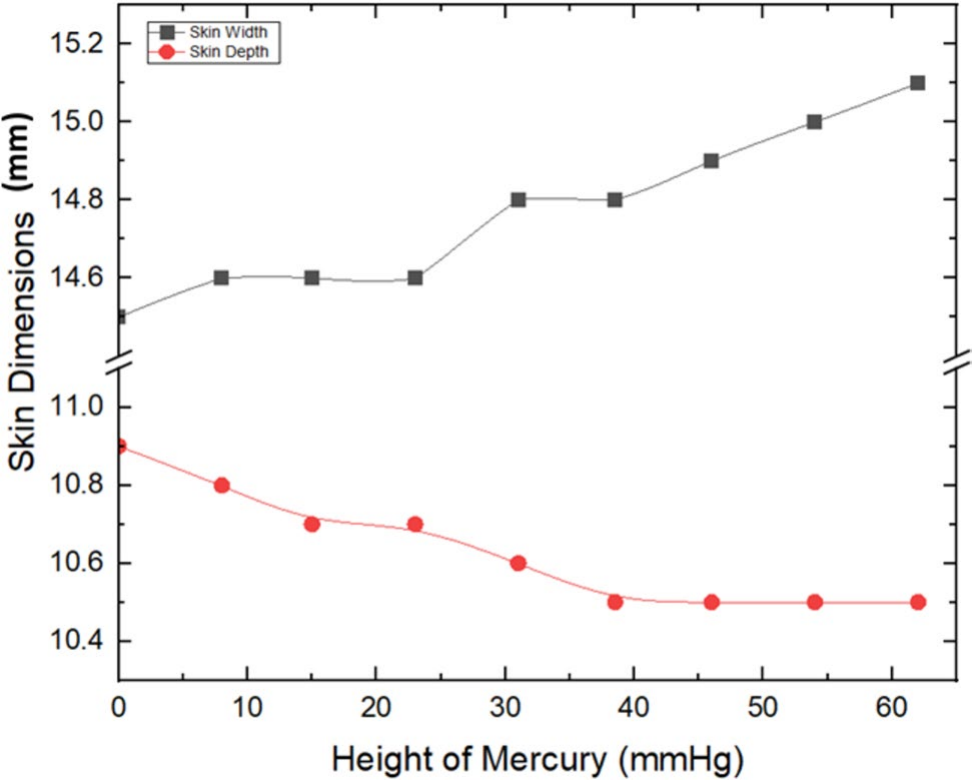
Skin width and depth monitoring against pressure

Skin dimensions’ monitoring against recorded pressure during the experiment is illustrated in Fig. 6. The monitoring of the skin width and depth throughout the experiment was crucial for ensuring that the pressure was being transmitted to the skin and not being absorbed by the balloon inside. Although slight changes in skin dimensions were observed, they were deemed to have no significant impact on the rectified RC.

The sensitivity analysis indicates that skin impedance is a critical parameter for accurate measurement of intrauterine pressure using sound waves. Since the impedance of a pregnant woman’s skin may vary due to factors such as hydration or tissue composition, the proposed method will need calibration in practical applications to account for these variations. This suggests that an individualized calibration step may be necessary in future device designs.

**Fig. 7:**
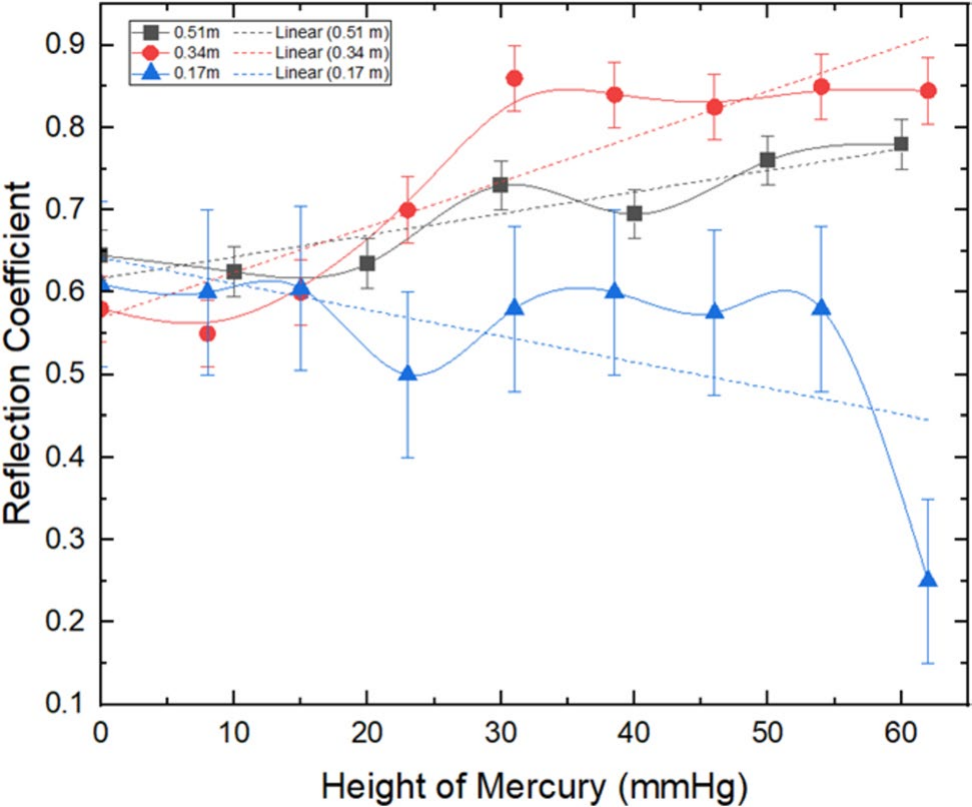
Reflection coefficient against pressure, at 4 kHz and 0.51 m, 0.34 m, and 0.17 m far from the skin model

**Fig. 8:**
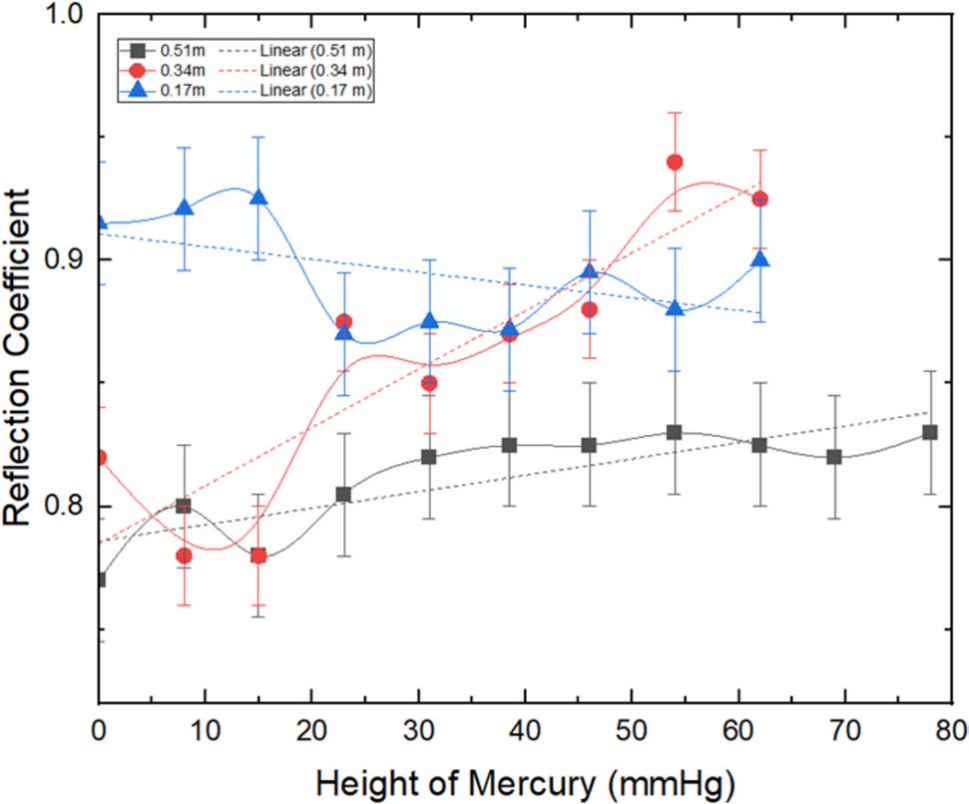
Reflection coefficient against pressure, at 20 kHz and **a** 0.51 m, **b** 0.34 m, and **c** 0.17 m far from the skin model

The relationship between internal pressure inside the skin model (measured in terms of the height of mercury (mmHg)) and the reflection coefficient (RC) of sound from it, at different distances; 170, 340, and 540 mm from the skin, and at a frequency of 4 kHz is displayed in Fig. 7. A corresponding plot at a frequency of 20 kHz is given in Fig. 8. The rectified RC was calculated using Eq. 1 (see ‘Methods’ section), since all recorded decibel values were in negative units. For both frequencies, a noticeable increase in the rectified RC was observed with a corresponding increase in the internal pressure. However, for a distance of 170 mm and for both frequencies, the relationship exhibited a slight adverse gradient. This might be due to an effect of any noise or discrepancies in the data leading to the negative slope. These findings illustrate that a distance of 170 mm was the least effective distance analyzed during the experimental testing, revealing the potential limitations associated with obtaining accurate measurements at very close distances. The maximum RC obtained in this study at frequencies of 4 and 20 kHz were 0.82 (with a standard deviation of 0.1) and 0.93 (with a standard deviation of 0.06), respectively. The outcomes from the analysis of the gradients obtained from Figs. 7 and 8 might suggest a correlation between the rectified RC and the pressure measurements.

Comparing the results in Figs. 6, 7, and 8 could be a suggestion that although there were slight variations in skin dimensions, they were too minimal to meaningfully affect the RC values. Therefore, it could be anticipated that the pressure-induced RC changes are more influenced by the material properties (such as stiffness, porosity, and acoustic impedance) rather than by physical deformation of the skin model.

The experimental data demonstrated that the RC exhibited a commensurate increase with the rise in the internal pressure. However, the results from the measurements conducted at the higher frequency of 20 kHz, depicted in Fig. 8, exhibited significantly greater RC values, ranging between 0.15 and 0.2, when compared to the RC values observed at 4 kHz. Although the overall standard deviation (SD) for 20 kHz was reported as 0.06, it represents an average across all distances and frequencies. At specific distances in Fig. 8, the SD is smaller, allowing for a clearer trend in RC values to be observed. The variation in error bars reflects the experimental conditions at different distances, but the overall trend remains consistent and supports the conclusions drawn. This finding indicates that more sound was reflected at the higher frequency, which may be attributed to the skin’s lower absorption rate of 20 kHz frequencies compared to 4 kHz, as well as the greater focusing ability of higher frequency sound, leading to an overall higher RC. This is because higher frequencies have shorter wavelengths, which allows them to focus more precisely on smaller areas, reducing dispersion and interference. In contrast, lower-frequency sound waves, such as 4 kHz, have longer wavelengths and are more likely to disperse over a wider area, leading to a lower RC. This increased focusing ability of higher frequency waves allows for better reflection detection and less interference from surrounding noise or materials, contributing to the higher RC values observed at 20 kHz.

It should be also noted that the higher directional properties of 20 kHz sound waves also minimize the effects of destructive interference, resulting in clearer and more accurate reflections. At higher frequencies, the number of wavelengths over a given distance increases, which helps reduce phase differences and destructive interference, further enhancing the precision of the RC measurements. This contrasts with the behaviour at 4 kHz, where the broader wavefront and longer wavelength can cause more interference and reduced accuracy.

Nevertheless, the RC obtained at 20 kHz had a narrower range than that obtained at 4 kHz, measuring only 0.1 at its widest, which may limit its practicality in distinguishing between different pressure levels. This narrow range is equivalent to an accuracy of approximately ±10 mmHg, which could have clinical implications in differentiating between strong and weak contractions. This observation highlights that the current model, while demonstrating a positive correlation, may not be optimal for use in a clinical setting. Further modifications may be necessary to enhance the design’s sensitivity and achieve more precise outcomes.

An additional factor of critical significance pertains to the specifications of the smartphone employed and its sound emission and detection limits. Specifically, at 20 kHz, the smartphone operates at the verge of its capacity for sound generation and detection. Consequently, its sensitivity towards higher frequencies may be adversely impacted, resulting in elevated uncertainties and noise. However, despite the presence of noise in the 20 kHz samples, the relevant features can still be extracted and exploited. Importantly, no evidence in the data suggests that the performance of a smartphone operating at 20 kHz is beyond its operational limits or yields suboptimal results due to the constraints of its capabilities.

In the analysis of the data, it was observed that the measurements obtained at 4 kHz tended to exhibit a larger standard deviation in comparison to the 20 kHz measurements. Specifically, the average spread for the RC values obtained at 20 kHz was 0.06, in contrast to the 0.1 standard deviation for 4 kHz, indicating a substantial disparity between the two frequencies. This outcome is most likely attributable to the directional properties of higher frequency sound waves, enabling most of the sound wave to be reflected from the centre of the skin. In contrast, at 4 kHz, some of the wave would be dispersed, even if it is perfectly aligned with the centre of the skin, since the wavefront is broader than the surface area of the skin’s centre. These findings highlight the benefit of higher-frequency sound in terms of its ability to differentiate between different pressure levels.

The results of the experiment revealed that there is an apparent optimal distance for each of the frequencies, as illustrated in Figs. 7 and 8, with the optimum distance for 20 kHz being shorter than that for 4 kHz. Specifically, the data showed that 20 kHz at a distance of 340 mm displayed the most extensive range of RC and did not plateau, rendering it the best distance for this frequency. In contrast, for 4 kHz, both 340 mm and 510 mm demonstrated comparable ranges of RC, but at a distance of 510 mm, there was no evidence of a plateau, indicating that this distance may be more effective for measurements.

The optimum distance between the smartphone and skin is dependent upon the frequency of the wave, which is in turn linked to the wavelength and its dissipation characteristics. A shorter wavelength might result in a more directional wave, making it easier to calibrate, especially when it acts in close proximity to the skin. Moreover, there may be a potential correlation between the phase difference and the number of wavelengths in the path. For instance, as the 20 kHz wave has a wavelength five times shorter than that of the 4 kHz wave, there are 20 wavelengths between the source and skin at 340 mm and 20 kHz, compared to just four wavelengths at 4 kHz. At a distance of 510 mm, the number of wavelengths for 4 kHz is six between the source and skin. When the number of wavelengths is an even integer, phase difference and destructive interference are minimized, resulting in maximum accuracy when recording the RC.

This could be an indication that the optimal distance can be estimated for each frequency based on the number of wavelengths, though additional data points may be required to determine the exact relationship between wavelength and distance. In summary, the optimal distance for recording the RC is dependent on the frequency of the wave and can be identified by considering the number of wavelengths, with shorter wavelengths and even numbers of wavelengths generally resulting in higher accuracy.

The occurrence of plateaus in the RC versus internal pressure curves suggests that the maximum RC value has been reached for a given distance and frequency. Regardless of the frequency, the plateau is observed at closer distances to the skin model, with the distance at which it occurs being proportional to the frequency. It is important to note that the plateau corresponds to the limit of reflected sound and is not observed at an RC value of 1.0 due to various losses. Further studies are necessary to elucidate the factors contributing to the onset and characteristics of the plateaus.

## Discussion

Previous research has explored non-invasive methods for estimating intrauterine pressure based on nonlinear parameters computed from electrohysterogram signals. While these studies have demonstrated feasibility, they often require complex equipment and setups. This study advances the field by leveraging widely accessible consumer technology, such as smartphones, to achieve similar objectives in a more practical and user-friendly manner [57]. The maximum reflected sound and RC values for a given distance and frequency are affected by various factors such as absorption, dissipation, and destructive interference. Absorption occurs when some of the soundwave energy is transferred to the new body as heat or friction upon impact, which is more pronounced for low-frequency waves than higher-frequency ones [58].

In the current study, it could be argued that the porosity was shown to directly impact the material’s acoustic impedance, defined as the product of its density and sound speed. Higher porosity increases compliance and reduces density, resulting in greater sound absorption and lower RC values. Lower porosity creates a denser, stiffer material with a higher acoustic impedance, leading to increased RC values. As internal pressure increases, the compression of voids within the material reduces the effect of porosity. This leads to a stiffening of the material, resulting in higher RC values across all porosity levels. This behaviour is reflected in the trends shown in Fig. 5, where RC values for both porosity levels converge at higher pressures. Experimental results had also shown that the maximum RC values obtained at frequencies of 4 and 20 kHz were 0.85 and 0.9, respectively. Absorption depends on the material properties of the medium the soundwave is interacting with, and as the skin model used is a close approximation of real skin, the generated values are likely to be reliable if replicated in vivo. However, the skin model’s pore distribution may differ from that of actual skin, which could alter how the skin interacts with the sound [40, 58, 59]. Future work could investigate dynamic porosity changes in real time or explore non-uniform porosity distributions to better simulate biological tissues.

It should be emphasized that at 4 kHz, the reflection coefficient (RC) was observed to be lower compared to 20 kHz. This is attributed to the longer wavelength of 4 kHz, which increases wave dispersion, leading to a broader wavefront. As a result, the sound waves are less focused, causing greater loss due to interference and lower overall reflection. In contrast, at 20 kHz, the shorter wavelength leads to more directional wave propagation, allowing for a sharper interaction with the skin model. This reduced dispersion enhances the RC values and increases the precision of pressure detection.

Therefore, in this study, the selection of 20 kHz was justified based upon the following reasons:

1. Directional properties: High-frequency waves (e.g., 20 kHz) have a smaller diffraction angle due to their shorter wavelength, which enables better focus on specific regions of the skin model. This minimizes wave scattering and ensures more precise reflection measurements.
2. Non-audibility: 20 kHz lies at the upper limit of human hearing and is effectively inaudible to most people. This characteristic makes it ideal for continuous or long-duration monitoring applications, as it avoids potential discomfort associated with audible sound frequencies.
3. Reduced environmental interference: Higher frequencies are less likely to overlap with background noise, which often occurs at lower frequencies (e.g., in the 2–8 kHz range). This contributes to a better signal-to-noise ratio and more accurate data collection.

Moreover, while 4 kHz demonstrated usability in the study, the broader wavefront and increased susceptibility to interference make it less suitable for high-precision applications. However, it may still be relevant in scenarios requiring greater penetration depth, as lower frequencies tend to propagate more effectively through denser media. The selection of 20 kHz thus balances the trade-offs between precision, non-audibility, and minimal interference, making it the preferred frequency for this study’s non-invasive monitoring approach. Finally, the 20 kHz frequency exhibited a narrower RC range (approximately 0.1), corresponding to an accuracy of ±10 mmHg, which aligns well with clinical requirements for distinguishing between strong and weak contractions.

The exclusion of sweat from the skin model could lead to small deviations in both the RC and plateau values. However, the impact of sweat on the experimental results is difficult to quantify, as individual variation in sweat production is known to exist. Sweat has a comparable acoustic impedance to that of water, with a value of 1.48 × 10^6^ *kg m*^−2^ *s*, which is lower than that of skin, resulting in a reflection fraction of 99.8% compared to skin’s 99.9%. The 0.1% difference is unlikely to have a significant effect on the accuracy of the measurements but could potentially lead to false negatives. Furthermore, the presence of sweat could increase the diffusivity of the reflection surface, leading to a further reduction in the detected RC. The exact impact of sweat on the RC and plateau values might require future studies to be accurately quantified.

Dissipation refers to the rate at which a wave loses its energy. For waves of equal amplitude and different frequencies, the higher-frequency wave will decay faster [15]. This leads to a lower RC when the wave has to travel further, as evidenced by Figs. 7 and 8. Dissipation affects the power of the wave, resulting in a lower RC value, which in turn makes it more difficult to distinguish the signal from noise, causing more errors as the distance from the skin increases. Due to the power fading to a level equivalent to the background noise, dissipation limits the maximum distance at which a measurement can be made from the skin.

In summary, destructive interference is a phenomenon that occurs when waves that are out of phase interfere with one another, leading to a reduction in their amplitude [60]. This would account for the lower RC values observed in Fig. 7 for the 4 kHz frequency at a distance from the skin model of 170 mm. The prevalence of destructive interference at this distance is due to the divide’s inability to effectively prevent soundwaves from crossing over to the reflected side, where they encounter reflected waves while out of phase, resulting in a diminished RC. Another potential contributing factor is wave diffraction, in which waves are bent around the edge of an obstacle, resulting in a reduction of the detected volume. However, this effect is likely to be minimal, as the gap between the divide and skin was kept at least twice as large as the largest wavelength. Further research might be required to investigate the relative contributions of these factors to the observed phenomenon.

In the case of using a single phone as both the speaker and the microphone, the same RC value was detected at every pressure level due to the phone picking up the sound directly from the speaker rather than measuring reflections. This issue cannot be addressed by using a sound barrier as it would impact the direction and focus of the sound waves [22]. Alternatively, a waveguide in the form of directional tubing could potentially be used. A waveguide in this context refers to a structure designed to direct and constrain the propagation of sound waves along a specific path, minimizing dispersion and interference. The goal would be to prevent external noise and unintended reflections that could distort the measurements. Unlike a wavelength shifter, the waveguide would ensure that the sound waves remain focused, reducing phase shifts and signal degradation. This approach would require careful calibration and further testing and could be explored in future research to optimize the detection of intrauterine pressure using sound waves. The optimal angle of reflection requires further investigation, as the experiment was conducted with a fixed reflection angle of 20° from the skin centreline. The angle should be kept small to avoid the direct travel of the wave from the speaker to the microphone, which could lead to an inflation of the reflected values, making them indistinguishable and leading to a recording of the incident wave instead. This occurs because the measurable parameter is the power or volume of the wave, and recording the incident wave directly renders all data useless. It is essential to determine the angle of reflection that provides maximum sensitivity to the reflected waves while avoiding the direct wave. Furthermore, the angle of reflection could potentially affect the shape of the reflected wave, which could have an impact on the RC values. Future studies could investigate the effect of different reflection angles on the RC values and determine the optimal angle for accurate and reliable measurements.

The results of the study indicated that a clear correlation existed between the RC and the internal pressure. It was also shown that the use of a conventional smartphone was sufficient for both emitting and detecting the required sounds to obtain accurate data, provided that two separate devices are used for each function—one dedicated to sound emission and the other for sound detection. This separation is crucial to prevent the direct path interference that would occur if a single device was used for both functions. Using two devices allows for clearer distinction between the emitted and reflected sound waves, ensuring that only the reflected signals are captured for analysis, thereby improving the accuracy and reliability of the data. This setup minimizes errors such as cross-talk or the detection of direct sound from the emitter, which would otherwise compromise the integrity of the pressure measurements.

In addition, the distance at which the smartphone is placed was an essential factor that impacts the quality of the data obtained and is frequency-dependent, with higher frequencies enabling closer measurements. Moreover, the angles of the phone must be carefully considered to ensure that the majority of the wavefront is reflected directly towards the microphone. Finally, the divide between the speaker and microphone is necessary to prevent any non-reflected sound from interfering with the reflected sound off the skin. However, the optimal compromise between the optimum distance and angle remains a critical trade-off for future studies and developments in this field.

The experimental setup used to model the skin and abdomen can be further improved to more accurately simulate real-world conditions. The fixed sides of the skin model to the wooden base could potentially impact how the skin stretches under pressure, and the direction of printing of the skin model could also play a role in this. Specifically, since the skin was printed with the x-y direction being its widest point and the z-axis in the direction of the curved dome, it could lead to a more pronounced z-direction elongation due to the skin being stronger in the x-y directions [61].

It should be clarified that there were some limitations and constraints associated with the equipment used and the conditions of the experimental environment. These limitations are elaborated below:

1. Equipment limitations

The equipment used in this study included consumer-grade smartphones (iPhone 12 and Samsung Galaxy S21) and the “Frequency Sound Generator” application. While these tools are accessible and cost-effective, they introduce inherent variability in frequency accuracy and sound wave generation. Calibration was conducted to mitigate these limitations, with observed frequency deviations of ±0.1 kHz at 20 kHz and ±0.05 kHz for the application. Although these deviations are minor, they may affect precision in applications requiring higher accuracy. Moreover, the smartphones lacked advanced control features, such as amplitude modulation, that could further enhance experimental flexibility. Laboratory-grade acoustic equipment with precise frequency generation and detection capabilities could provide more accurate and reliable data.

2) Environmental constraints

The experiments were conducted in a controlled laboratory environment to minimize external interference. However, the possibility of background noise and acoustic interference impacting the results cannot be completely excluded. While physical partitions were used to isolate the experimental setup, these measures do not replicate the ideal conditions of an anechoic chamber. Future experiments should consider using advanced noise-cancellation techniques or conducting tests in acoustically isolated environments to eliminate external noise and improve the fidelity of the results. Additionally, temperature and humidity conditions were not explicitly controlled during the experiments. These environmental factors can influence the acoustic properties of both the air medium and the physical models, potentially affecting the reflection coefficient (RC). Accounting for these variables in future studies will enhance result consistency.

3) Physical model limitations

The physical models used in this study were fabricated using FDM and were designed with uniform porosity and elasticity to simulate the properties of biological tissues. While effective for this purpose, these models lack the inherent heterogeneity and anisotropic characteristics of actual biological tissues. In real tissues, porosity and elasticity vary spatially and dynamically, particularly under pressure, which could significantly influence acoustic reflection. The material composition of the models also does not replicate the biochemical properties of human skin or subcutaneous tissues, which can impact sound wave propagation and absorption. As a result, the current models provide an approximation but may not fully represent real-world biological conditions.

4) Experimental setup and data analysis constraints

The study focused on static pressure conditions to simulate steady-state intrauterine pressures. While this approach allowed for controlled measurements, it does not account for the dynamic, cyclic pressure variations characteristic of labour contractions. Future studies should explore dynamic pressure simulations to better mimic physiological scenarios. In addition, the analysis of RC data assumed uniform wave propagation and reflection, which may not hold true in real-world applications due to the heterogeneity of tissues. Such complexities introduce variability not accounted for in this study and warrant further investigation.

## Conclusion

This study successfully demonstrated the potential of using sound waves emitted by a smartphone for non-invasive intrauterine pressure (IUP) measurement, providing an innovative, cost-effective alternative to traditional diagnostic methods. By correlating the reflection coefficient of sound waves to internal pressure changes, the research highlighted the practicality of using frequencies of 4 kHz and 20 kHz, with 20 kHz proving superior due to its non-audible nature and reduced measurement error. The FEM simulations supported the hypothesis that changes in IUP can be detected through sound wave reflections from the abdominal model. The experimental results indicated that, while 20 kHz offered better precision for sustained monitoring, there was still a need for further refinement of the technique to improve sensitivity and reliability, especially in real-world clinical settings. Future work should focus on optimizing the model for more accurate, patient-specific measurements and validating the approach in vivo to ensure it can be used effectively for labour diagnostics.

## Data Availability

The datasets used and/or analyzed during the current study available from the corresponding author on reasonable request. All data generated or analyzed during this study are included in this published article.
